# The effect of Co-Q10 on allergic rhinitis and allergic asthma

**DOI:** 10.1186/s13223-021-00534-5

**Published:** 2021-03-20

**Authors:** Qixue Du, Wei Meng, Seyyed Shamsadin Athari, Renzhong Wang

**Affiliations:** 1 Shandong University of Traditional Chinese Medicine, Jinan, 250001 Shandong China; 2 Department of Otolaryngology, Jinan Municipal Hospital of Traditional Chinese Medicine, Jinan, 250001 Shandong China; 3 Department of Otolaryngology, Affiliated Hospital of Shandong University of Traditional Chinese Medicine, Jinan, 250001 Shandong China; 4 Department of Immunology, School of Medicine, Zanjan University of Medical Sciences, Zanjan, Iran

**Keywords:** Coenzyme, Th2, Signaling, Allergy, Cytokine, Immunoglobulin

## Abstract

**Background:**

Allergic asthma is an inflammatory disease resulting from continued or intermittent allergen exposure, and allergic rhinitis can be trigger of asthma. The main mechanism of these disease is allergic reaction and immune response dysregulation. Co-Q10 is an enzyme cofactor in mitochondria can control asthma and allergic rhinitis symptoms. In the present study, we determined that the CoQ10-induced anti-allergic effects were mediated by up-regulation of Nrf2.

**Methods:**

Animal models of allergic rhinitis and allergic asthma were produced and treated with Co-Q10, Co-Q10 and O-3, Co-Q10 and Mg-S. Bronchoalveolar lavage fluid was collected from animal models, and IL-4, 5, 13, INF-y, Eicosanoids, IgE, EPO, and histamine production were measured. Also, COX-2, CCL24, CCL11, Nrf2, Eotaxin, Cytb, COX1 and ND1 genes expressions and histopathology were studied. BALf's cells were collected by tracheostomy and used in slide producing by cytospine. Cytokines, Eicosanoids, IgE, EPO, and histamine were measured by ELISA method. Gene expression was done by Real-time PCR.

**Results:**

Co-Q10 with two supplementation (Mg-S and O-3) modulate MRC, BALf eosinophils, eosinophilic inflammation related genes (eotaxin, CCL11 and CCL24), peribronchial and perivascular inflammation, EPO, type 2 cytokines (IL-4, 5 and 13), IgE, histamine, Cyc-LT and LTB4 as main allergic bio-factors. Importantly, Co-Q10 treatment increased Nrf2 expression and Nrf2 induced antioxidant genes, glutathione redox and inhibited inflammation, oxidative stress injury, Th2 cytokines production and attenuated allergic inflammatory responses.

**Conclusion:**

Nrf2 is activated in response to allergen, induces resistance against the rhinitis and asthma development and plays an essential role in broncho-protection. Co-Q10 increases the Nrf2 expression and the Nrf2 over-expression has strong effect in control of type2 cytokines, allergic mediators and inflammatory factors that lead to harnessing of allergy and asthma.

**Graphic abstract:**

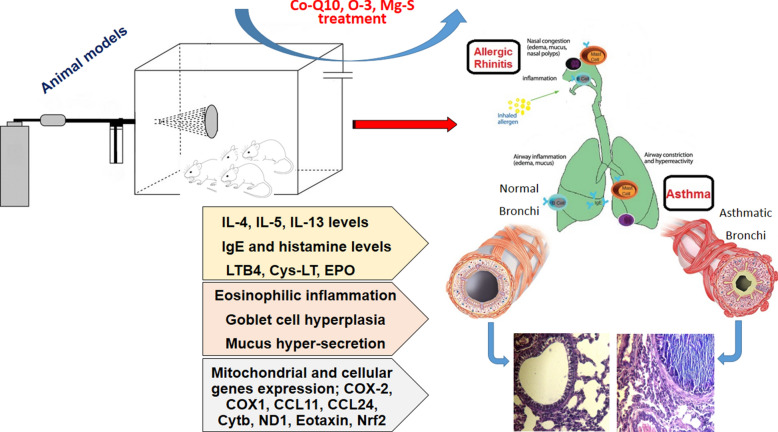

## Introduction

Asthma is a complex chronic inflammatory disease of the bronchi. The heterogeneity of airway inflammation during asthma indicates there are different mechanisms involved. It is recognized that asthma is a chronic inflammatory disease resulting from continued or intermittent allergen exposure, and allergic rhinitis can be trigger of asthma. The main mechanism of these disease is allergic reaction and immune response dysregulation. Genetic predisposition and environmental factors are main triggers for initiation of allergic reactions, and in the atopic patients (who can have expressed allergic related genes), when allergen inters to the body, that can beginning allergic reactions [1, 2].

Allergic diseases substantially, affect up to 25% of the population in industrialized societies and are a major public health problem [3]. The allergic diseases pathophysiology is complex. The most common allergic diseases include allergic rhinitis, conjunctivitis, allergic asthma, atopic dermatitis, food allergy, and anaphylaxis. In the allergic conditions of respiratory system, when allergens are entering to the lung by inhalation, it can take place in the respiratory tract, and subsequently, is transported from the lumen to the mucosa through epithelial cells. In the mucosa, allergen is internalized by dendritic cells (DCs), and after processing in DC cytoplasm, the DCsmove to T-cell areas of draining lymph nodes, interact with naive T cells and present epitops of allergen on MHC class II molecules to the T cells. If people have allergic related genes, type 2 cytokines will be dominant and lead to the polarization of Th cells to produce Th2 cells. The Th2 releases more type 2 cytokines and initiates allergic mechanism. IL-4 secreted from Th2 forced B cells to immunoglobulin isotype class-switching to the IgE. IgE as a hallmark of allergic sensitization is fundamental antibody in patients with allergic diseases. Produced IgE will be bound to IgE receptors [high-affinity receptors for IgE (Fc*ε*RI)] on surface of mast cells and basophils. Allergen upon re-exposure binds to the IgE receptor on the surface of mast cells and basophils and leads to release of allergic bio-factors such as histamine, serotonin, prostaglandin, proteases, and etc., which handling allergic reaction symptoms such as allergic rhinitis [2–4].

The other key cytokines IL-5, and IL-13, have main role in allergic asthma pathophysiology, which amplify and maintain Th2-driven allergic inflammation. IL-5 increases the eosinophils production and it can lead to eosinophilic inflammation in lung that is main problem in treatment of asthmatic patients. The potential of eosinophils to cause tissue injury is observed in asthma. Increased IL-13 results in increased mucus secretion in the airway; in asthma attacks, this results in bronchial obstruction [2, 5].

Co-Q10, which is structurally similar to vitamin K, has three biological functions. It enhances mitochondrial ATP for energy production, provides antioxidant effects, and enhances cell membrane stabilization. Co-Q10 also known as ubiquinol-10, is an endogenous enzyme cofactor in mitochondria and lysosomes that catalyzes proton/electron translocation and protects mitochondria from free radical damage may play a role in preventing programmed cell death or apoptosis [6–8]. However, mitochondria signaling has important effects in asthma pathophysiology [2] and using Co-Q1 can control asthma and allergic rhinitis symptoms. Nrf2 in airway epithelial and smooth muscle cells protects against the pro-inflammatory and oxidizing effects, thus protects in asthma. Nrf2-mediated anti-oxidative stress effect is critical for maintaining epithelial barrier integrity and prevents allergen entrance. However, in the presence of ROS, Nrf2 is stabilized due to the disruption of Keap1-mediated repression, and in nucleus, activates cytoprotective target genes by binding to AREs or ROS-responsive elements as a Nrf2/smallMaf heterodimer. Also, Nrf2 regulates the glutathione and thioredoxin-dependent antioxidant systems by modulation of subunits of the glutathione-synthesizing enzyme glutamate-cysteine ligase, and regulation of the thioredoxin-associated factors expression such as thioredoxin reductase 1. On the other hand, disruption of epithelial tight junctions leads to impaired barrier function that allows inhaled allergens to pass more easily into the bronchi wall and interact with immune cells, thereby increases susceptibility to respiratory allergens. Aldehyde oxidase (AOX)1 functions downstream of Nrf2 in the bronchi epithelial barrier formation, and therefore, Nrf2/AOX1 pathway alleviate asthma by enhancing airway epithelial barrier [9, 10]. We used Co-Q10 to investigate a potential asthma therapy, which targets the Nrf2 pathway. In the present study, we determined that the CoQ10-induced anti-allergic effects were mediated by up-regulation of Nrf2 and the induction of anti-inflammation defense mechanisms.

## Materials and methods

### Animal models and treatment schedule

Female 7–8 week-old BALB/c mice were raised 1 week under standard conditions in laboratory animal house to adaptation and all experiments were done according to the Laboratory Animal Care ethical guidelines. 90 mice were divided into nine experimental groups to produce allergic rhinitis and asthma models that include: negative control group (healthy mice) that was sensitized and challenged with PBS; the remained eight groups had been sensitized and challenged with OVA. four groups as allergic asthma that includes: asthma mice with no treatment, asthma mice treated with Co-Q10 (50 mg/kg orally), asthma mice treated with Co-Q10 (50 mg/kg orally) and O-3 (500 mg/kg orally), asthma mice with Co-Q10 (50 mg/kg orally) and Mg-S (MgSO4; 10 mg/kg orally), four groups as allergic rhinitis that includes: allergic rhinitis mice with no treatment, allergic rhinitis mice were treated with Co-Q10, allergic rhinitis mice were treated with Co-Q10 and O-3, allergic rhinitis mice were treated with Co-Q10 and Mg-S [11, 12].

### Sensitization and administration

**Fig. 1:**
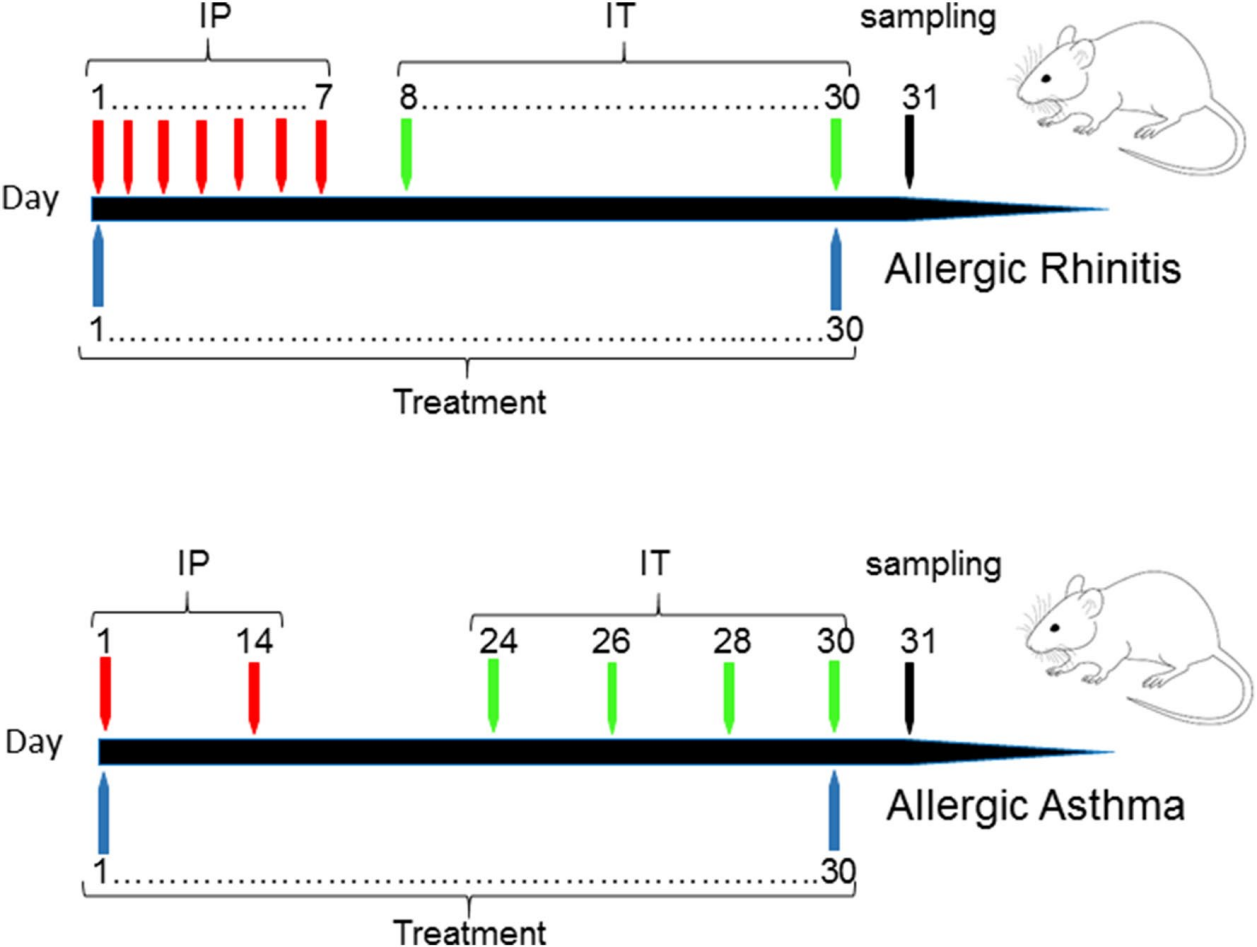
Allergic rhinitis and asthma animal model. BALB/C mice were sensitized by OVA with alum on days 1 and 14 (IP), and challenged by OVA solution on days 24, 26, 28 and 30 (IT) to produce asthma mouse model. For rhinitis model, BALB/C mice were sensitized by OVA with alum on days 1–7 (IP), and challenged by OVA solution on days 8–30 (IT). Treatment was done on days 1–30 and sampling on days 31

Mice sensitization and challenging were shown in Fig. 1 that described previously [4, 13]. Briefly, for asthma model production, mice were sensitized with 20 µg OVA solution with 50 µl alum adjuvant on days 1 and 14 by IP injections and challenged with 1%OVA solution by nebulizer for 30 min on days 24, 26, 28, 30 by IT. For allergic rhinitis model production, briefly, 0.3 mg OVA and 30 mg alum was injected IP every day for seven times. Then, 2 mg OVA in 20 µl normal saline was instilled via IT daily for seven times (Fig. 1). Every day IT OVA administration was continuous to maintain allergic rhinitis conditions. The treated groups received treatment by oral administration on days 1–30 and at the day 31, the BALf, blood and lung tissue samples were taken by euthanizing of mice.

### BALf’s cell counting

After anesthetization of mice, BALf was collected from the trachea of mice via intubation (from healthy and four asthmatic groups), then centrifuged and the supernatant collected for cytokine and chemokine level analysis. BALF cells were fixed to the slide using cytospine and stained with Giemsa stain to determine the percentage of EOs present.

### Cytokines

The levels of IL-4, 5, 13 and INF-γ were measured by specific ELISA kits according to the manufacturer’s instructions (R&D, USA).

### Eicosanoid levels

LTB4 and Cys-LT were assayed in BALf supernatant using ELISA kits (Cayman Chemical, Ann Arbor, USA) according to the manufacturer’s instructions.

### Serum Ig

After centrifuging blood samples, serum was separated, and then total IgE level was measured by ELISA (BD Biosciences, USA) method.

### Histamine level

In the blood samples, the level of histamine was determined in serum by ELISA Kits (Biocompare, USA).

### QrtPCR

After centrifuged BALf, the cells suspension has been stored and then, RNA was extracted from BALf cells using TRI reagent. According to extracted RNA, the cDNA was synthesized using a cDNA synthesis kit. At least, the target genes expressions were studied using SYBR Green Master Mix (Bio-Rad) by specific primers that were shown in Table 1. GAPDH and Actb as housekeeping gene, were used as internal reference gene.

**Table 1:** Used primers sequences

Gene	5′-3′	Primer
COX-2	Forward	ACCAGCAGTTCCAGTATCAGA
	Reverse	CAGGAGGATGGAGTTGTTGTAG
CCL24	Forward	AGGCAGTGAGAACCAAGT
	Reverse	GCGTCAATACCTATGTCCAA
CCL11	Forward	GGCTTCATGTAGTTCCAGAT
	Reverse	CCATTGTGTTCCTCAATAATCC
Nrf2	Forward	TCTCCTCGCTGGAAAAAGAA
	Reverse	AATGTGCTGGCTGTGCTTTA
Eotaxin	Forward	CTGCTCACGGTCACTTCCTT
	Reverse	GGGGTCAGCACAGATCTCTT
GAPDH	Forward	TGTTCCTACCCCCAATGTGT
	Reverse	GGTCCTCAGTGTAGCCCAAG
Actb	Forward	AGAAGCTGTGCTATGTTGCTCTA
	Reverse	TCAGGCAGCTCATAGCTCTTC
ND1	Forward	ATTACTTCTGCCAGCCTGACC
	Reverse	GGCCCGGTTTGTTTCTGCTA
Cytb	Forward	GGCTACGTCCTTCCATGAGG
	Reverse	TGGGATGGCTGATAGGAGGT
COX1	Forward	ATCACTACCAGTGCTAGCCG
	Reverse	CCTCCAGCGGGATCAAAGAA

### Mitochondria isolation and MRC genes expression

The lungs were placed in ice-cold homogenization buffer with pH 7.4 containing 10 mM HEPES, 70 mM sucrose, 2% fatty acid-free BSA, 200 mM mannitol, 1 mM EGTA, and protease inhibitor cocktail Set III (50 µl/g tissue) and minced over ice. Lung tissue was homogenized and centrifuged at 2000×*g* and 4 °C for 15 min. The supernatant was centrifuged at 17,800×*g* at 4 °C for 15 min. The resulting supernatant was re-suspended in 5 ml ice-cold homogenization solution and centrifuged at 17,800×*g* at 4 °C for 15 min. The supernatant was discarded and re-suspended in 2 ml ice-cold buffer (homogenization buffer without BSA) and stored. The mitochondrial cDNA was synthesed and expression of complex I, III and IV as mitochondrial-encoded electron transport chain genes were done [14, 15].

### EPO

EPO level was determined in BALf according to previous study [16]. Briefly, 1 ml substrate solution, containing 0.1 mM o-phenylenediamine dihydrochloride, 0.1%Triton X-100, and 1 mM hydrogen peroxide in 0.05 M Tris(hydroxymethyl)aminomethane hydrochloride, were added to 1 ml BALf and incubated at 37 °C for 30 min. The reaction was stopped by adding 0.5 ml 4 M sulfuric acid and the absorbance was read at 492 nm.

### Histological study of lung

Lung tissues of the four asthmatic groups and healthy group were taken and fixed with formalin, then cut in slide sections for staining with H&E and AB-PAS. The produced slides were evaluated under the light microscopy for eosinophil infiltration around bronchi and vessels (peribronchial and perivascular inflammation), goblet cell hyperplasia and mucus hypersecretion [4].

### Statistical analysis

The SPSS version 19 was performed for statistical analyses using ANOVA and a Dunnett post hoc test. The data were shown as the mean ± SD of at least three independent experiments. Pearson’s method was used for correlation analysis. The graphs were shown with GraphPad prism and P < 0.05 was considered significant.

## Results

### BALf’s cells

**Fig. 2:**
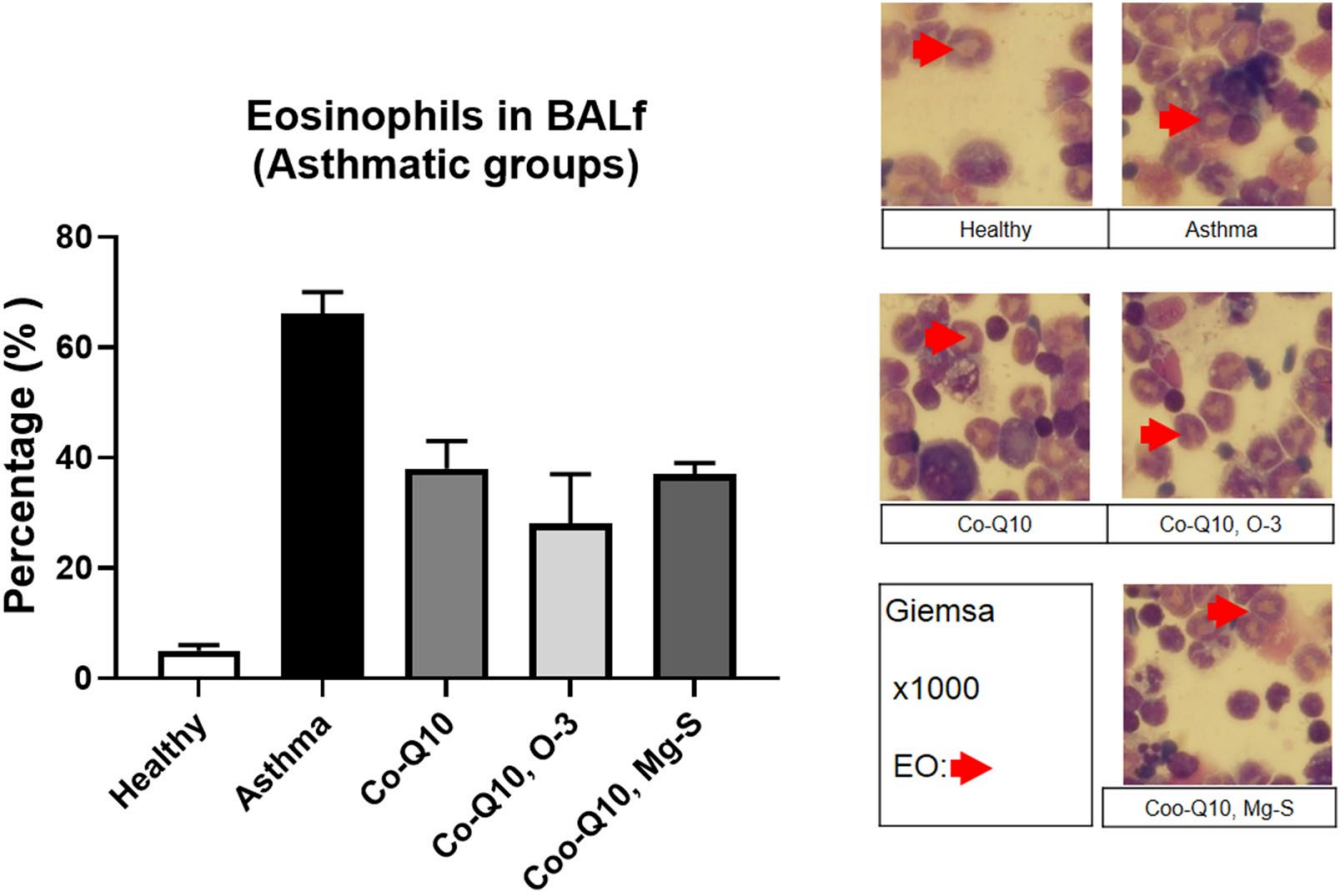
Eos percentage. The percentage of eosinophil was assessed in BALf of studied groups on days 31 by cytospine. The Eos was shown with ×1000 by giemsa staining

The eosinophils percentage in the BALf of asthmatic groups were counted. Asthmatic group had increased the eosinophils percentage in the BALf compared to healthy group on days 31 (66 ± 4 versus 5 ± 1%, *P* < 0.05). The eosinophil percentage were significantly decreased by three treatment (Co-Q10: 38 ± 5%, Co-Q10, O-3: 28 ± 9%, Co-Q10, Mg-S: 37 ± 2%) on day 31 compared to non-treated asthmatic group (*P* < 0.05) (Fig. 2).

### Cytokines

**Fig. 3:**
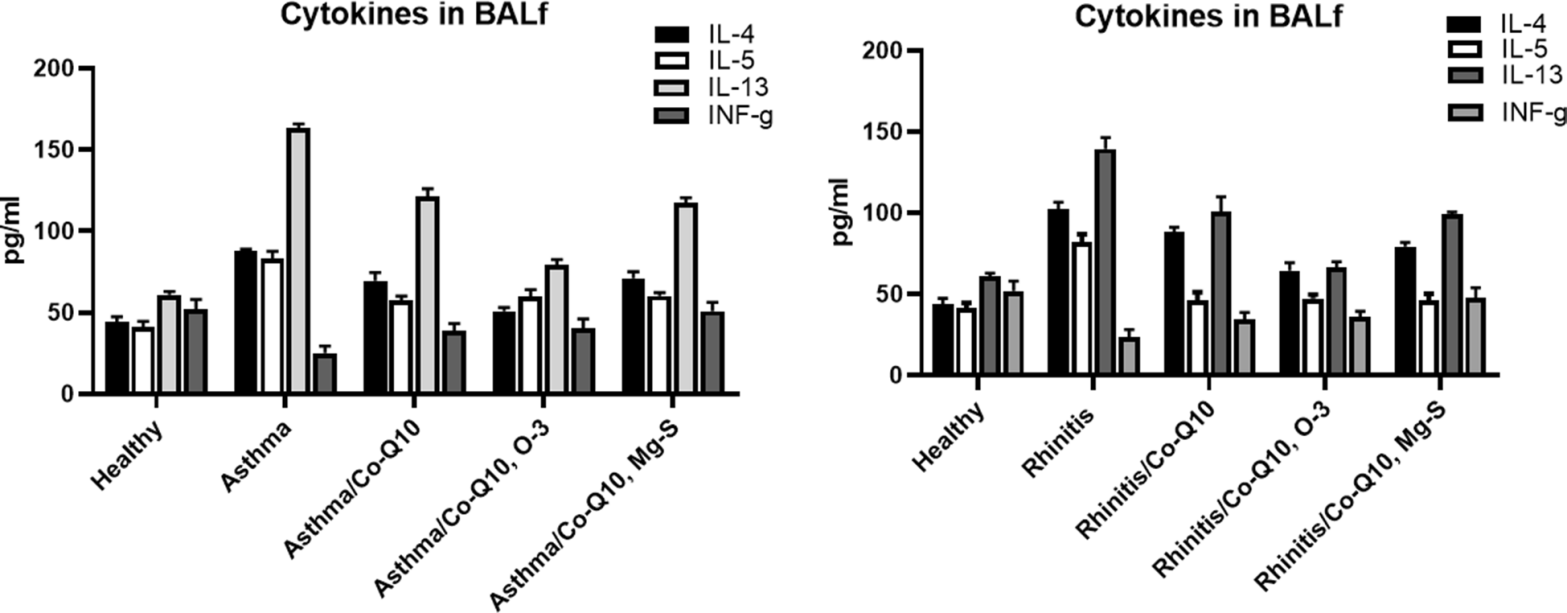
Cytokines levels. The levels of IL-4, IL-5, IL-13 and INF-γ in BALf were measured in all groups of allergic rhinitis and allergic asthma on day 31

The levels of IL-4 (87.54 ± 1.45 and 102.0 ± 4.65 pg/ml respectively), IL-5 (83.43 ± 4.23 and 82.09 ± 4.76 pg/ml respectively), and IL-13 (163.65 ± 2.34 and 139.43 ± 6.98 pg/ml respectively) were increased in the asthma and rhinitis animals on day 31 compared with those seen in the healthy animals (IL-4: 44.03 ± 3.37, IL-5: 41.23 ± 3.43, IL-13: 6087 ± 1.99 pg/ml) and a reverse trend was found in IFN-γ (asthma 25.11 ± 4.32 and rhinitis 23.64 ± 4.67 groups compared with healthy group: 51.98 ± 6.11 pg/ml) (*P* < 0.05). In the three treated groups of asthma and also, allergic rhinitis (Co-Q10 received groups, Co-Q10 and O-3 received groups, Co-Q10 and Mg-S received groups), reverse trend was found and significantly reduced IL-4, IL-5, IL-13, and restored the IFN-γ levels (*P* < 0.05) (Fig. 3).

### Eicosanoid levels

**Fig. 4:**
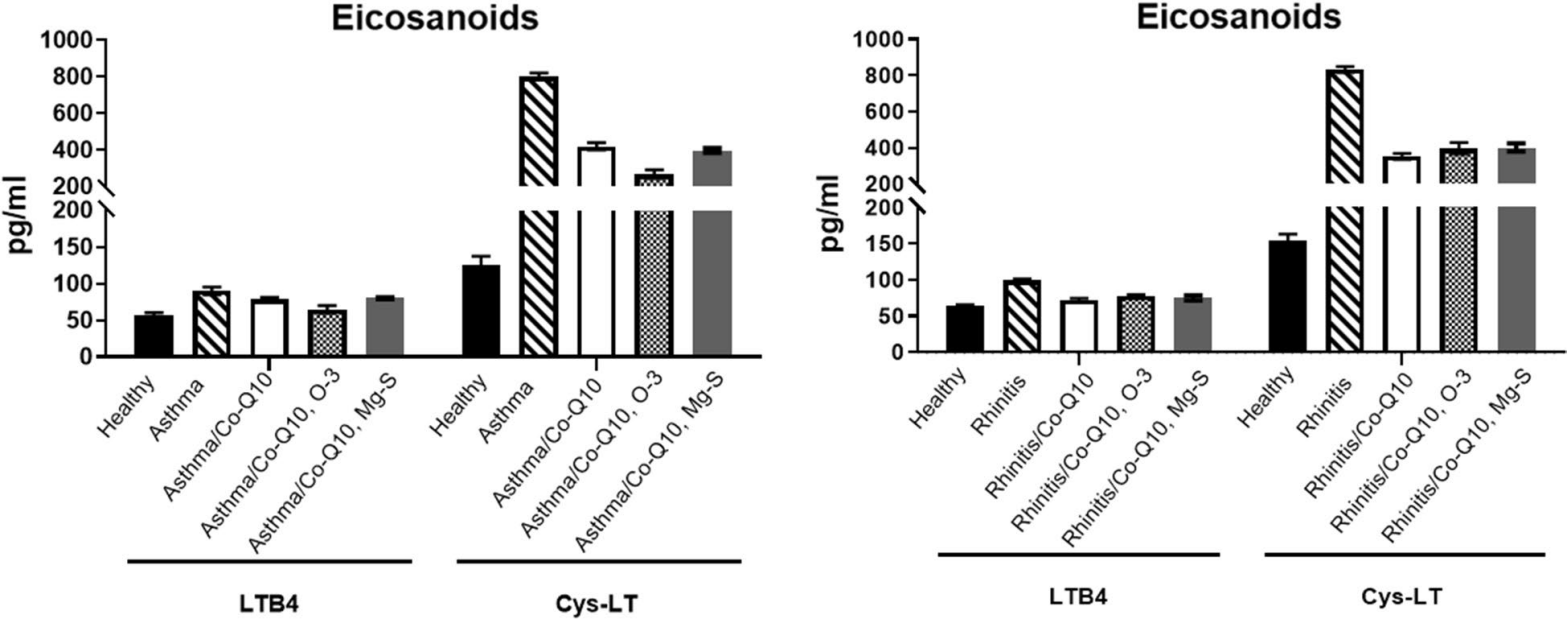
Cys-LT and LTB4 levels. The levels of cysteinyl leukotriene (Cys-LT) and leukotriene B4 (LTB4) were measured on days 31 in allergic rhinitis and asthma groups

The level of LTB4 was significantly decreased in Co-Q10 (78.43 ± 2.83 and 72.03 ± 2.21 pg/ml respectively) and Co-Q10, O-3 (65.04 ± 4.87 and 77.54 ± 1.95 pg/ml respectively) treated asthma and rhinitis and Co-Q10, Mg-s (75.23 ± 3.60 pg/ml) treated rhinitis groups compared to non-treated asthma (90.11 ± 5.27 pg/ml) and rhinitis (99.65 ± 1.54 pg/ml) groups (*P* < 0.05). The level of Cys-LT in BALf was significantly decreased in all three treated asthma and rhinitis groups compared to non-treated asthma and rhinitis groups (*P* < 0.05) (Fig. 4).

### Serum IgE

**Fig. 5:**
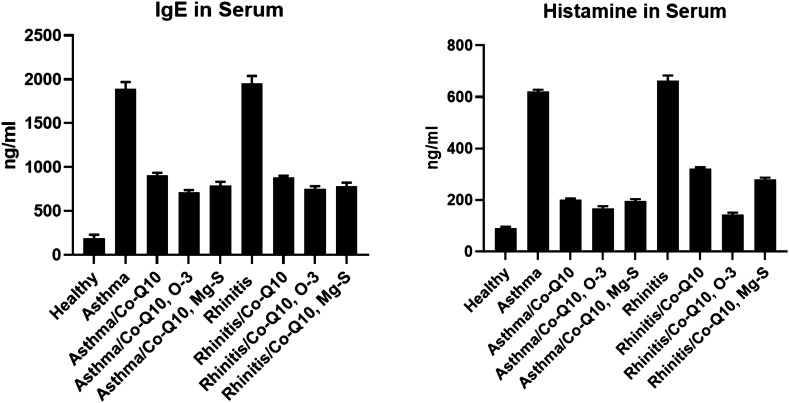
IgE and histamine levels. The levels of total histamine and IgE were measured in the serum of all groups

Asthma and rhinitis groups significantly enhance total IgE level in serum (*day* 1892.4 ± 76.9*,* 1952.6 ± 85.9*)* compared to healthy group (186*.*9 ± 44.1) *(P* < 0.05). Three treatments in all asthma (Co-Q10: 903.4 ± 32.1; Co-Q10, O-3: 713.2 ± 24.3; and Co-Q10, Mg-s: 789.4 ± 43.6 ng/ml) and rhinitis (Co-Q10: 883.5 ± 19.4; Co-Q10, O-3: 748.3 ± 34.6; and Co-Q10, Mg-s: 783.5 ± 40.0) groups reduced significantly total IgE level in serum in compared with non-treated groups (Fig. 5).

### Histamine level

The histamine level in serum was significantly increased in asthma and rhinitis groups on day 31 (620.01 ± 8.43 and 663.72 ± 19.98 ng/ml respectively) compared to negative control group (90.21 ± 6.32 ng/ml) (*P* < 0.05). The three treatment in all asthma and rhinitis groups reduced significantly histamine level in serum in compared with non-treated groups. The histamine level decreasing was significant in Rhinitis group that was treated with Co-Q10, O-3 (142.97 ± 8.3 ng/ml) (*P* < 0.05) in compare other two treated groups (Co-Q10: 320.89 ± 6.9 and Co-Q10, Mg-S: 279.93 ± 7.4 ng/ml) (Fig. 5).

### Qrt-PCR

**Fig. 6:**
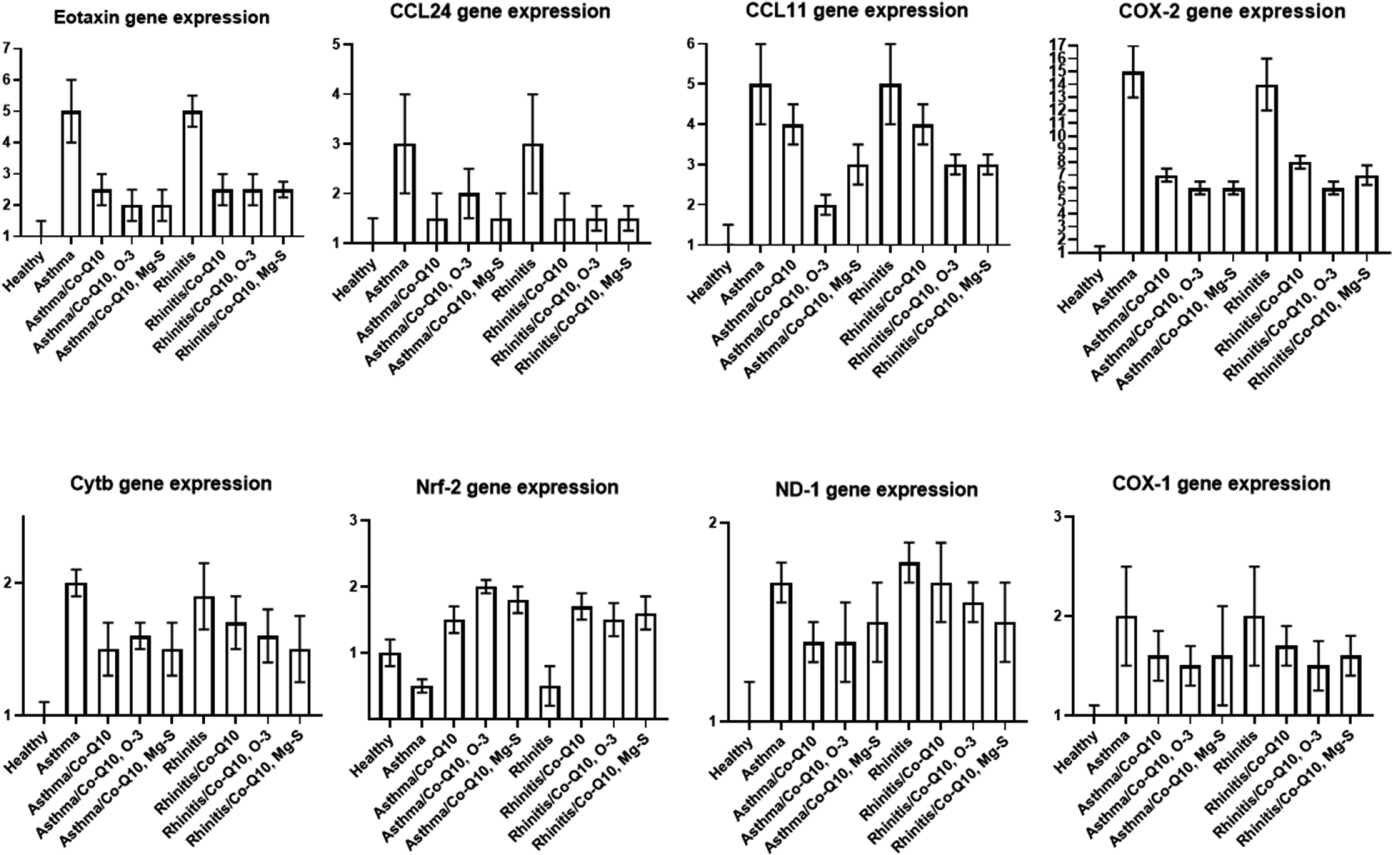
QrtPCR. Effect of three treatments on the mRNA gene expression of COX-1, COX-2, eotaxin, CCL11, CCL24, ND-1, cytb and Nrf-2 in BALf cells were determined by Qrt-PCR

In the asthma and rhinitis groups that were treated with Co-Q10, Co-Q10, O-3 and Co-Q10, Mg-S, mRNA expression of Eotaxin, and COX-2 were significantly decreased (*P* < 0.001). In the treated groups, Co-Q10 could reduce ND-1 gene expression in asthma group and Co-Q10, O-3 could reduce COX-1 gene expression in asthma group. In the asthma and rhinitis groups that were treated, the expression of CCL11 in Co-Q10 treated asthma and rhinitis groups, CCL24 in Co-Q10, O-3 treated asthma group, and Cytb in Co-Q10, Co-Q10, O-3 and Co-Q10, Mg-S treated rhinitis groups were decreased but the decreasing was not significant and other groups were significantly decreased these genes expression. Nrf2 expression was decreased in asthma and rhinitis groups and with treatment (Co-Q10, Co-Q10, O-3 and Co-Q10, Mg-S), the expression was increased significantly in asthma and rhinitis groups (Fig. 6).

### EPO

**Fig. 7:**
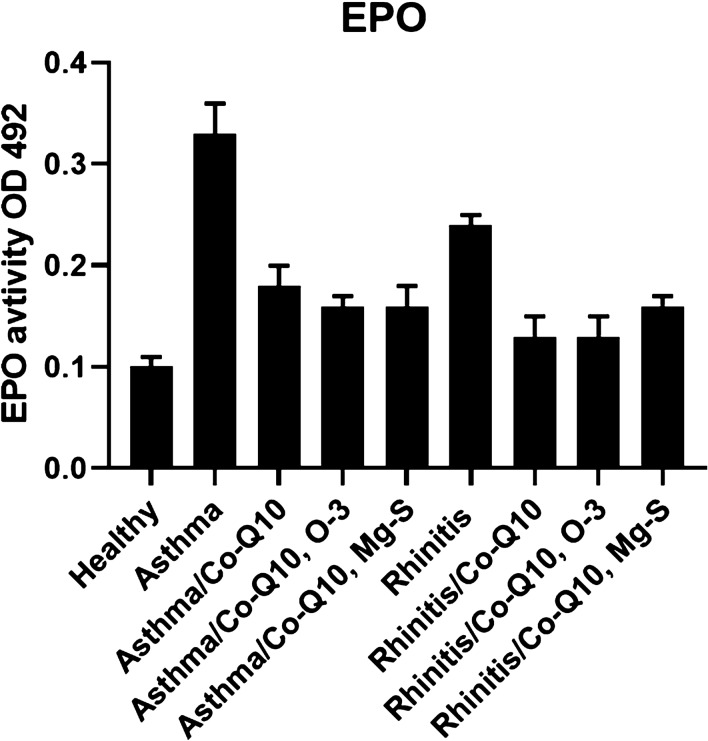
EPO. The EPO activity was studied in all treated and non-treated groups

The EPO activity was significantly decreased in three treated asthma (Co-Q10 treated asthma: 0.18 ± 0.02, Co-Q10, O-3 treated asthma: 0.16 ± 0.01, Co-Q10, Mg-S treated asthma: 0.16 ± 0.02) and rhinitis (Co-Q10 treated rhinitis: 0.13 ± 0.02, Co-Q10, O-3 treated rhinitis: 0.13 ± 0.02, Co-Q10, Mg-S treated rhinitis: 0.16 ± 0.01) groups compared to non-treated asthma (0.33 ± 0.03) and non-treated rhinitis groups (0.24 ± 0.01) (Fig. 7) (*P* < 0.05).

### Histopathology

**Fig. 8:**
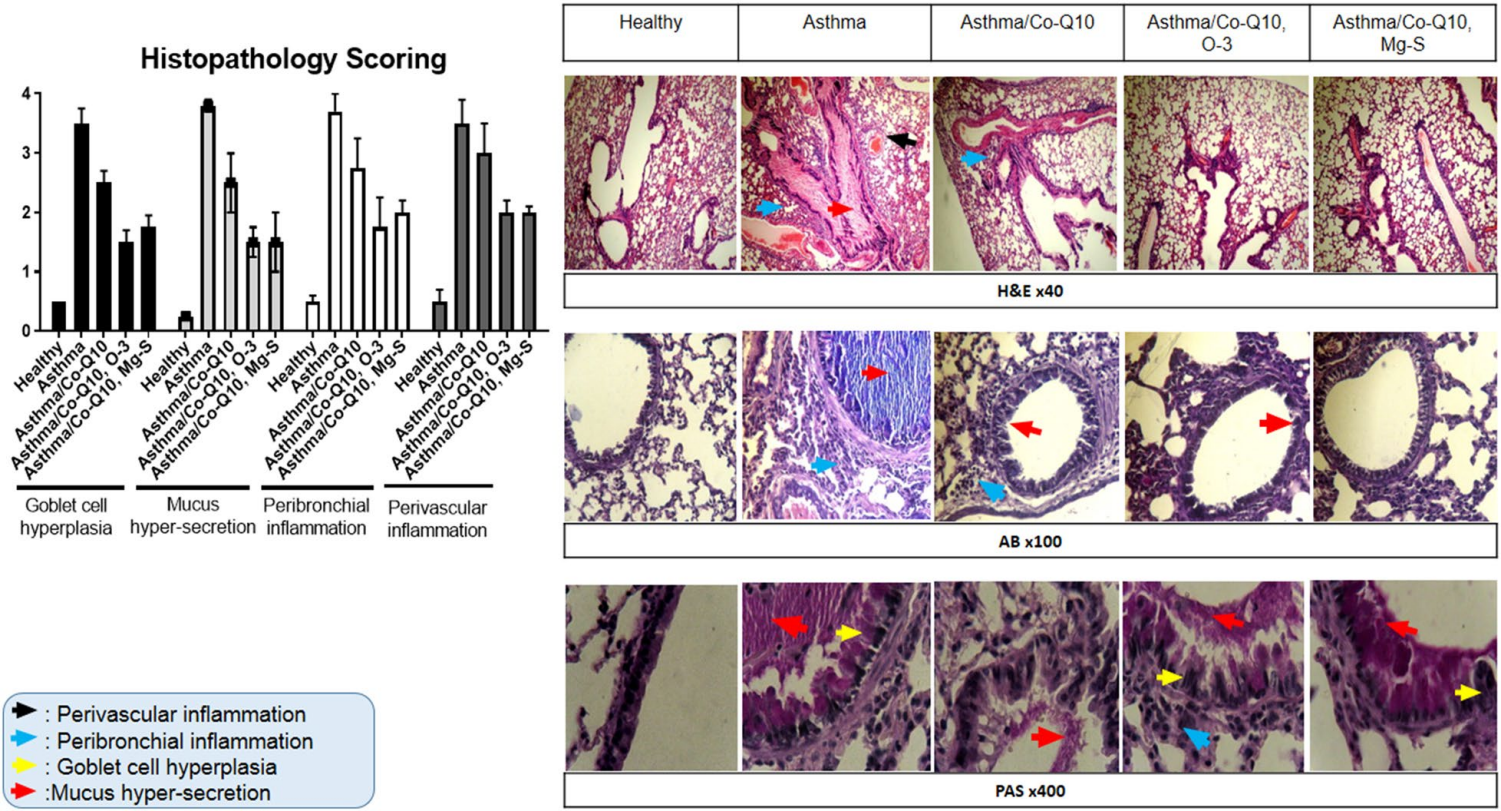
Histopathology sections. Histological sections of lungs of all groups were stained with H&E, PAS and AB on day 31. The peribronchiolar and perivascular inflammation, goblet cell hyperplasia and mucus hypersecretion were evaluated in all groups. Eosinophil infiltration around bronchi and vessels, secretion of the mucus and hyperplasia of the goblet cells were analyzed

Perivascular inflammation, peribronchial inflammation, Goblet cell hyperplasia and mucus hyper-secretion were significantly decreased in the bronchi of asthmatic group that were treated with Co-Q10, O-3 and Co-Q10, Mg-S. Goblet cell hyperplasia and mucus hyper-secretion were significantly decreased in the bronchi of asthmatic group that were treated with Co-Q10. Perivascular and peribronchial inflammation were decreased in asthma group that was treated with Co-Q10 compared to asthma group but not significant (Fig. 8).

## Discussion

Bronchial inflammation is principal focus in asthma pathogenesis and asthma treatment. Disbalance between free oxygen radicals and anti-oxidative defense is important in control of airway inflammation. Free oxygen radicals have strong contribution in the pathogenesis of allergic asthma. The modulation of oxidation and antioxidative defense represents new therapy in complex management of asthma. Co-Q10 is one of the main supplementations that has important effect in antioxidative defense [17]. In this study, we observed that treatment with Co-Q10 and other additive supplementations can control allergic rhinitis and allergic asthma pathophysiology related factors and Co-Q10 may be useful in the treatment of allergic diseases. Combined anti-inflammatory therapy is preferred in order to minimize the manifestations of adverse reactions. In this study we used Co-Q10 with two part of combined therapy.

Co-Q10 is an essential factor that contributes to the development of mitochondrial pathology, MRC function, and allergic inflammation. Dietary disorders and hereditary factors contribute to the development of acquired Co-Q10 deficiency, which lead to mitochondrial abnormalities in the asthmatic patients [18, 19]. Mitochondrial abnormalities are related with oxidative stress that causes damage to a variety of biomolecules in membranes, proteins, and DNA, which contribute to asthmatic airways injury [18, 19]. Co-Q10 an integral part of the mitochondrial electron transport chain, scavenges free radicals and has anti-inflammatory properties. Corticosteroids as effective drugs in the asthma treatment inhibit inflammatory cytokine production. In asthma, Co-Q10 was associated with reduction of the corticosteroid dose [19, 20]. In this study, Co-Q10 treatment reduced main type 2 cytokines (IL-4, 5 and 13) and enhanced main type 1 cytokine (INF-γ) in asthma and rhinitis groups. Therefore, Co-Q10 can control inflammation in airway and allergic reactions in upper and lower airways and modulate immune response in lung. This treatment significantly controls IgE, histamine, Cyc-LT and LTB4 as main allergic bio-factors and allergic response mediators.

Although the importance of Co-Q10 in mitochondrial function is widely recognized, Co-Q10 low level in serum of asthmatic patients has been reported [17]. However, the oxidative phosphorylation and ATP production are dependent upon the MRC complexes function and it was reported that Co-Q10 deficiency has pathologic effect on MRC function. Co-Q10 is an essential MRC component and MRC dysfunction is frequently associated with deficiency of Co-Q10, which may lead to mitochondria degradation. Co-Q10 deficiency also contributes to increased reactive oxygen species generation and in result, inflammation processing [18]. In this study, we found that Co-Q10 can modulate MRC and reactive oxygen species related genes. This effect was emphases when it was with supplementary treatment. Also, eosinophilic inflammation related genes such as eotaxin, CCL11 and CCL24 genes expression have been controlled and harnessed by Co-Q10 treatment and especially when Co-Q10 was used with Mg-S and O-3. These treatments can control mucus secretion and goblet cell hyperplasia, which are main obstruction factors of airway. On the other hand, when Co-Q10 has Mg-S and O-3 as additive supplementation, peribronchial and perivascular eosinophilic inflammation and also eosinophil present in BALf were decreased significantly. Three treatment protocols can reduce EPO and therefore, may be important treatment in control of eosinophilic injury in bronchi. In the presented investigation, effect of Co-Q10 on airway inflammation was survived and the effect of Co-Q10 on eosinophilic injury in airways was not studied and needs future investigations.

Co-Q10 represents an important member of the antioxidative potential and plays a decisive role in the cell energy production and in the scavenger activity. In the study by Gazdík et al., it was reported that Co-Q10 concentrations was decreased in plasma of asthmatic patients compared with healthy volunteers [17]. Therefore, increased level of Co-Q10 can have protective effect on pulmonary functions.

The Nrf2 is a redox-sensitive basic leucine zipper transcription factor and involved in the transcriptional regulation of antioxidant genes. Nrf2 disruption increases expression of the Th2 cytokines and enhances asthma severity. Nrf2 is activated in response to allergen, induces transcriptional of antioxidant genes that provide resistance against the asthma development [21]. It has been reported that Nrf2 deficiency enhances bronchial inflammation in asthma. Disruption of Nrf2 also leads to eosinophils increases in lung tissues, airway remodeling, AHR, goblet cell hyperplasia, and level of Th2 cytokines. The Nrf2 signaling plays an essential role in broncho-protection [22]. In our study, Co-Q10 treatment can increased Nrf2 expression and significantly enhanced Nrf2 gene activation. Therefore, Co-Q10 treatment control inflammation and in result, oxidative stress injury in lung.

Nrf2 expression was associated with glutathione redox and the duration of asthma, the Nrf2 pathway is disrupted. Decreased Nrf2expression in asthmatic lung tissue associated with increased inflammation and steroid resistance [23]. In addition to, Nrf2-deficiency up-regulates IL-33 response and administrates allergy. Nrf2 activation suppresses IL-33 releasing and attenuates bronchial inflammation [24]. So, activation of Nrf2 expression leads to control of Th2 cytokines and upper hand of these cytokines (IL-33), and can be attenuate allergic and inflammation related agents and allergic rhinitis and asthma.

Co-Q10 as supplementary agent, has anti-inflammatory and antioxidant effects. Co-Q10 increases the Nrf2 expression. The Nrf2 over-expression controls allergic and inflammatory factors. On the other hand, the Nrf2 regulates the antioxidant related enzymes and increased anti-oxidative activity of this system. In the COVID-19 pandemy, one of the main dangerous complications of SARS-Cov2 infection is ARDS that increases morbidity and mortality of COVID-19 positive patients. ARDS is caused by pulmonary inflammation, and damage. ARDS may be suppressed by controlling of inflammation and cell damage by increasing anti-inflammatory and antioxidant factors. Co-Q10 supplementation with other anti-COVID-19 drugs, might be beneficial on patients with ARDS [22, 25]. Moreover, two interactional factors, oxidative stress and inflammation involve in ARDS and activation of Nrf2 can control oxidative stress and inflammation in ARDS [22]. Therefore, Nrf2 activation with Co-Q10 and other treatments can be effective drug in control and treatment of ADRS in COVID-19. According to our search, there was no research about effect of Co-Q10 on ARDS of the COVID-19 mouse model. Since, there was no related research, it is suggested that future researches have focused on effect of Co-Q10 on ARDS and COVID-19 lung inflammation.

ARDS caused by SARS-Cov2 is followed by alveolar cells damage and lung fibrosis and the host immune response (with cytokine storm) and inflammation is a crucial determinant of disease outcome. Cytokine storm is associated with pulmonary inflammation, and respiratory system damage and ARDS in molecular glance, is presented by cell death and production of pro-inflammatory cytokines. Regulating of this pathway may be useful in treatment of COVID-19 patients with harnessing of ARDS. Nrf2 as cytoprotective transcription factor can boost endogenous cellular defenses, control inflammation, restore redox homeostasis and also facilitate tissue repair. Nrf2 binds to antioxidant response elements, and regulates proteins production that are involved in cellular redox homeostasis, detoxification, cell damage repair, and metabolic balance. Importantly, activated Nrf2 is involved in preserving lung architecture in response to inflammation, and has strong therapeutic effects ARDS. Moreover, Nrf2 plays a role in the inflammation resolution by repressing IL1-β, IL6, TNF-α and other pro-inflammatory cytokines (cytokine storm inhibition). Also, Nrf2 induces tissue repair genes, anti-oxidative protein, CD36 as scavenger receptor, and IL-17D as protector against viral infections [25]. In COVID-19, SARS-CoV-2 infection depends the host cell factors ACE2 and TMPRSS2. Virus binds to the cell via ACE2 receptors and spike protein must be cleaved by serine protease TMPRSS2 for enter to the cell. Nrf2 as a regulator of respiratory viral infections susceptibility, down-regulates ACE2 (a surface receptor) and TMPRSS2 activates the spike protein for SARS-Cov-2 entry into host cells. Nrf2 has ability to impede viral entry, slows viral replication, and reduces inflammation and mortality in respiratory viral infection. It may prove suitable treatment for COVID-19 cases [26]. Nrf2 activation reduce lung cells damage in COVID-19 patients and Inhibits virus penetration. Moreover, the Nrf2 activation improves phagocytosis and clearance through a mechanism is independent of the intracellular-antioxidant glutathione. Nrf2 regulates GSH via controlling and modulatory release of γ-glutamyl cysteine ligase and also, induces anti-oxidant enzymes such as NQO1, HO-1, SODs, Grx1, and Trx1 production [27]. In this study, we hypothesis that pharmacological Nrf2 activation in the context of SARSCov-2 infection can inhibit ARDS, protect lung cells, act as anti-inflammation, harness cytokine storm, inhibit viral integration, control oxidation stress by ROS scavenging, and present other cytoprotective effects. Therefore, in COVID-19, Nrf2 not only prevents of viral iterance, but also, harnessing ARDS and hyper-inflammation by control cytokines storm and oxidative stress, and repairs lung tissue that needs more investigation in future.

Co-Q10 as a main supplementation has anti-inflammatory effect when used with Mg-S and O-3 as combined therapy. It can activate Nrf2 expression in response to allergen, induces resistance against the asthma development and plays an important role in protection on upper and lower airways against allergic response and pathology, control inflammation and moreover, ARDS. Because, Co-Q10 has anti-inflammatory and antioxidant effects, might be beneficial for ARDS by controlling of lung inflammation and cell damage in the COVID-19 patients.

## Data Availability

Not applicable.

## Abbreviations

AB-PAS: Alcian blue-periodic acid Schiff
Actb: Beta actin
ARDS: Acute respiratory distress syndrome
ATP: Adenosine triphosphate
BALf: Broncho alveolar lavage fluid
CCL: C–C motif chemokine ligand
CD: Cluster of differentiation
Co: Coenzyme
COX: Cytochrome C oxidase
Cytb: Cytochrome B
EO: Eosinophil
EPO: Eosinophil peroxidase activity
GAPDH: Glyceraldehyde 3-phosphate dehydrogenase
H&E: Hematoxylin and eosin
Ig: Immunoglobulin
IL: Interleukin
IP: Intraperitoneal
IT: Inhalation administration
Mg-S: Magnesium sulfate
MRC: Mitochondrial respiratory chain
ND: NADH dehydrogenase
Nrf: The nuclear factor erythroid 2—related factor
O-3: Omega-3
OVA: Ovalbumin
PBS: Phosphate-buffered saline
Qrt: Quantitative real-time
SARS–Cov: Severe acute respiratory syndrome-corona virus
